# Audit and Feedback to Ordering Providers to Reduce Inappropriate C. difficile Testing and Hospital Onset C. difficile Rate

**DOI:** 10.1017/ash.2024.185

**Published:** 2024-09-16

**Authors:** Elise Martin, Vanessa Kung, Jody Feigel, Kristin Nagaro, Deanna Buehrle

**Affiliations:** VA Pittsburgh Healthcare System; Pittsburgh VA Hospital

## Abstract

**Background:** Inappropriate Clostridioides difficile (C. difficile) laboratory testing is common in hospitals and leads to over diagnosis, unnecessary treatment, and elevated hospital onset C. difficile infection (HO-CDI) metrics. Diagnostic stewardship is essential to avoid inappropriate testing, but limited data exists on optional interventions. **Methods:** A diagnostic stewardship intervention targeting CDI testing comprised of education and prospective audit with feedback was performed a VA facility (inpatient, outpatient, and long-term care units). Education on appropriate indications for CDI testing was provided in pre-intervention (9/2022 to 5/2023) and intervention periods (6/2023 to 12/2023). During the intervention period, all CDI tests (positive or negative) were audited after completion in real-time by an Infectious Diseases physician and feedback was given to ordering providers and/or their supervising physician (if trainee) for all tests not meeting an appropriate indication. Appropriate indication was defined as ≥3 liquid stools in 24 hours or symptoms of fulminant disease. Testing was considered inappropriate if no clinical symptoms, patient received laxatives within 48 hours, test was performed for test-of-cure or within 7 days of a prior test with no clinical change, or delayed testing in patients with diarrhea on admission. The rate of HO-CDI per 10,000 bed days of care (BDOC) per LabID event was compared during the pre-intervention and intervention periods, and ordering appropriateness was compared for all tests and hospital onset tests before (3/2023-5/2023) and after (6/2023-12/2023) feedback was performed. **Results:** After starting audit and feedback, HO-CDI rate decreased from 3.92 per 10,000 BDOC to 0.99 per 10,000 BDOC (p=0.03). HO-CDI rate among tests that were inappropriate was 2.19 and 0.80 per 10,000 BDOC during the pre-intervention and intervention periods, respectively (p=0.40). Average overall tests per month decreased from 37.8 to 28.1 after the intervention. Rate of all inappropriate tests decreased from 16.25 to 7.96 per 10,000 BDOC (p=0.04) and rate of hospital onset inappropriate tests trended toward decrease from 9.29 to 4.77 tests per 10,000 BDOC (p=0.07). The most common reasons for inappropriate testing were < 3 episodes of diarrhea in 24 hours (54% pre-intervention, 65% intervention) and laxative use (57% pre-intervention, 45% intervention). No cases of delayed testing leading to worsened disease were identified during the intervention. Cost savings for decreased tests were estimated at $150-300 per month. **Conclusion:** An intervention comprised of education and real-time audit and feedback of all CDI tests obtained at a VA facility resulted in decreased inappropriate testing and reduced the rate of HO-CDI.

